# ANCA-associated vasculitis in childhood: recent advances

**DOI:** 10.1186/s13052-017-0364-x

**Published:** 2017-05-05

**Authors:** Marta Calatroni, Elena Oliva, Davide Gianfreda, Gina Gregorini, Marco Allinovi, Giuseppe A. Ramirez, Enrica P. Bozzolo, Sara Monti, Claudia Bracaglia, Giulia Marucci, Monica Bodria, Renato A. Sinico, Federico Pieruzzi, Gabriella Moroni, Serena Pastore, Giacomo Emmi, Pasquale Esposito, Mariagrazia Catanoso, Giancarlo Barbano, Alice Bonanni, Augusto Vaglio

**Affiliations:** 1 Dialysis and Transplantation Policlinico IRCCS Fondazione San Matteo and University of Pavia, Pavia, Italy; 2 Nephrology Unit, University Hospital, Parma, Via Gramsci 14, 43126 Parma, Italy; 3 Nephrology Unit, Spedali Civili, Brescia, Italy; 4 Pediatric Nephrology Unit, Meyer Children’s University Hospital, Florence, Italy; 5 Internal Medicine, San Raffaele Hospital, Milan, Italy; 6 Policlinico IRCCS Fondazione San Matteo, University of Pavia, Pavia, Italy; 7 Division of Rheumatology IRCCS Ospedale Pediatrico Bambino Gesù, Rome, Italy; 8 Nephrology, Istituto G. Gaslini, Genoa, Italy; 9 Department of Medicine and Surgery, University of Parma, Parma, Italy; 10 Nephrology and University of Milano Bicocca, Milan, Italy; 11 Nephrology Unit, Fondazione IRCCS Ca’ Granda Ospedale Maggiore Policlinico, Milan, Italy; 12 Institute for Maternal and Child Health-IRCCS Burlo Garofolo, Trieste, Italy; 13 Internal Medicine, University of Firenze, Florence, Italy; 14 Rheumatology, Arcispedale S Maria Nuova, Reggio Emilia, Italy

**Keywords:** ANCA, Vasculitis, Glomerulonephritis, Childhood, Renal failure, Autoimmunity

## Abstract

Anti-neutrophil cytoplasmic antibody (ANCA)-associated vasculitides are rare systemic diseases that usually occur in adulthood. They comprise granulomatosis with polyangiitis (GPA, Wegener’s), microscopic polyangiitis (MPA) and eosinophilic granulomatosis with polyangiitis (EGPA, Churg-Strauss syndrome). Their clinical presentation is often heterogeneous, with frequent involvement of the respiratory tract, the kidney, the skin and the joints. ANCA-associated vasculitis is rare in childhood but North-American and European cohort studies performed during the last decade have clarified their phenotype, patterns of renal involvement and their prognostic implications, and outcome. Herein, we review the main clinical and therapeutic aspects of childhood-onset ANCA-associated vasculitis, and provide preliminary data on demographic characteristics and organ manifestations of an Italian multicentre cohort.

## Background

Primary systemic vasculitides comprise a group of disorders characterised by the presence of inflammation affecting the blood vessel wall, with resulting tissue ischemia and necrosis. Systemic vasculitides are usually classified based on the size of the predominantly involved vessels into large-, medium- and small-vessel vasculitides. The most common large-vessel vasculitides are Takayasu arteritis and giant cell arteritis; examples of medium-sized vessel vasculitis are polyarteritis nodosa and Kawasaki disease; finally, small-vessel vasculitides include immune-complex mediated or hypersensitivity forms (e.g. cryoglobulinemic vasculitis, IgA vasculitis/Henoch-Schönlein purpura) and anti-neutrophil cytoplasmic antibody (ANCA)-associated vasculitis. All of these forms, except for Kawasaki disease and IgA vasculitis, occur more frequently in the adult age and are extremely rare in childhood. Nevertheless, their recognition, correct diagnosis and appropriate treatment is crucial as they may be life- or organ-threatening [1].

ANCA-associated vasculitides (AAVs) are multisystemic diseases and include microscopic polyangitiis (MPA), granulomatosis with polyangiitis (GPA, formerly Wegener’s granulomatosis) and eosinophilic granulomatosis with polyangiitis (EGPA, formerly Churg-Strauss syndrome). According to the revised Chapel Hill Consensus Conference Nomenclature of vasculitides, AAV is defined as a necrotising vasculitis with few or no immune deposits, predominantly affecting small blood vessels (i.e. capillaries, venules, and arterioles), associated with the presence of circulating autoantibodies (ANCA) that are usually directed against myeloperoxidase (MPO) or proteinase 3 (PR3). PR3-ANCA account for the majority of ANCA with cytoplasmic immunofluorescence patterns (C-ANCA) and are commonly associated with GPA (60-80% of the cases), whereas MPO-ANCA usually match ANCA with perinuclear immunofluorescence pattern (P-ANCA) and are more common in patients with MPA (80-90% of the cases) or EGPA (35-40% of the cases) [2]. Data on AAV in paediatric populations are scarce, although recent cohort studies- mainly focusing on GPA and MPA and including up to 230 cases [3]- have greatly contributed to define the clinical phenotype of paediatric AAV. Conversely, studies on paediatric EGPA are limited to case reports or small retrospective case series. Finally, clinical trials and long-term outcome studies on all paediatric AAV forms are lacking, except for retrospective analyses focusing mainly on renal involvement. Herein, we provide an overview of AAV in children, particularly focusing on clinical features, kidney involvement, treatment and outcome.

### Definitions and classification criteria

In 1990 the American College of Rheumatology (ACR) proposed classification criteria for several (adult-onset) systemic vasculitides, including GPA and EGPA [4, 5]. The vasculitis working group of the Paediatric Rheumatology European Society (PReS) proposed the first classification criteria of vasculitis in children, endorsed by the European League against Rheumatism (EULAR) in 2006 [6]. The general classification for childhood vasculitides was based- as it is in adult patients- on the vessel size. The small-vessel vasculitides were then subcategorised into granulomatous and non-granulomatous (Table 1). These proposed modifications were mainly based on a literature review and not formally validated.

**Table 1:** Classification of childhood-onset vasculitis based on the EULAR/PReS endorsed consensus criteria [6]

I *Predominantly large-vessel vasculitis*
• Takayasu arteritis
II *Predominantly medium-sized vessel vasculitis*
• Childhood polyarteritis nodosa • Cutaneous polyarteritis • Kawasaki disease
III *Predominantly small-vessel vasculitis*
(A) GRANULOMATOUS • Wegener’s granulomatosis • Churg-Strauss syndrome (B) NON-GRANULOMATOUS • Microscopic polyangiitis • Henoch-Schönlein purpura • Isolated cutaneous leucocytoclastic vasculitis • Hypocomplementic urticarial vasculitis
IV *Other vasculitides*
• Behçet disease • Vasculitis secondary to infection (including hepatitis B associated polyarteritis nodosa), malignancies, and drugs, including hypersensivity vasculitis • Vasculitis associated with connective tissue diseases • Isolated vasculitis of the central nervous system • Cogan syndrome • Unclassified

In 2008, at the Ankara Consensus Conference, a joint effort of the EULAR, PReS and Paediatric Rheumatology International Trials Organisation (PRINTO) societies validated the EULAR-endorsed criteria and defined the classification criteria for the four main paediatric vasculitides (IgA vasculitis, GPA, polyarteritis nodosa and Takayasu arteritis). These classification criteria were based on a collection of data of 1398 children from different centres. GPA was the only form of AAV whose criteria were defined by the EULAR/PReS/PRINTO joint committee. Three of the following six criteria were required to classify paediatric vasculitis as GPA: histopathology (granulomatous inflammation within the arterial wall or in the perivascular or extravascular area), upper airway involvement (chronic purulent or bloody nasal discharge or recurrent epistaxis/crusts/granulomata, nasal septum perforation or saddle nose deformity, chronic or recurrent sinus inflammation), laryngo-tracheo-bronchial stenoses (subglottic, tracheal or bronchial stenosis), pulmonary involvement (chest x-ray or CT showing the presence of nodules, cavities or fixed infiltrates), ANCA positivity by immunofluorescence or ELISA (P-ANCA/MPO-ANCA or C-ANCA/PR3-ANCA) and renal involvement (proteinuria > 0.3 g/24 h or >30 mmol/mg of urine albumin/creatinine ratio on a spot morning sample, haematuria or red blood cell casts in the urinary sediment or ≥2+ on dipstick, or necrotising pauci-immune glomerulonephritis) (Table 2) [7, 8]. As compared with the ACR criteria for adult GPA, the EULAR/PReS/PRINTO criteria mainly differed for the addition of CT for a better characterisation of lung lesions and for the inclusion of more specific features for respiratory tract complications.

**Table 2:** Final EULAR/PRINTO/PReS criteria for granulomatosis with polyangiitis (GPA, Wegener’s) [8]

Criterion	Glossary	Sensitivity (%)	Specificity (%)
1-Histopathology	Granulomatous inflammation within the wall of an artery or in perivascular or extravascular area	54	99.6
2-Upper airway involvement	Chronic purulent or bloody nasal discharge or recurrent epistaxis/crusts/granulomata Nasal septum perforation o saddle nose deformity Chronic or recurrent sinus inflammation	83	99
3-Laryngo-tracheo-bronchial involvement	Subglottic, tracheal or bronchial stenoses	22	99.8
4-Pulmonary involvement	Chest x-ray or CT showing the presence of nodules, cavities or fixed infiltrates	78	92
5-ANCA	ANCA positivity by immunofluorescence or by ELISA (MPO/p or PR3/c ANCA)	93	90
6-Renal involvement	Proteinuria >0.3 g/24 ore or > 30 mmol/mg of urine albumin/creatinine ratio on a spot morning sample Haematuria or red blood cell casts: >5 red blood cells/high power field or red blood cell casts in the urinary sediment or ≥2+ on dipstick Necrotising pauci-immune glomerulonephritis	65	69.6
*Childhood-WG EULAR/PRINTO/PRES* *Ankara 2008 classification*	At least three of the six following criteria: Histopathology Upper airway involvement Laryngo-tracheo-bronchial stenosis Pulmonary involvement ANCA positivity Renal involvement	93.3	99.2

ANCA, anti-neutrophil cytoplasmic antibody; WG, Wegener granulomatosis; EULAR, European League Against Rheumatism; PReS, Paediatric Rheumatology European Society; PRINTO, Paediatric Rheumatology International Trials Organisation

No specific criteria for childhood-onset MPA and EGPA have been formulated. In adults, different sets of criteria have been proposed for EGPA, including the Lanham’s criteria (asthma, eosinophil count >1.5x10^9^/L and vasculitis involving two or more organs) [9] and the 1990 ACR criteria (asthma, paranasal sinus abnormalities, eosinophilia, lung infiltrates, peripheral neuropathy and extravascular eosinophils on biopsy- with at least four of these criteria required for the classification of vasculitis as EGPA) [5]. Conversely, MPA has no defined classification criteria in adults either. In 2007, Watts et al. developed a four-step algorithm to permit classification of MPA, GPA, EGPA and polyarteritis nodosa. This algorithm was adopted by the European Medicines Agency (EMA) as a practical tool for clinical trials and clinical study of adult AAV and also for GPA and MPA in peadiatric patients [10, 11]. No formal validation of the EMA algorithm for EGPA is available for children; therefore, the diagnosis of EGPA in childhood still relies on the empirical translation of the adult classification systems.

### Epidemiology

AAV has been mainly reported in adults. During the last 25 years, studies on the epidemiology of AAV in the adult age have been reported from Europe, Japan, USA, New Zealand and Australia. The overall annual incidence rates of AAV in Europe are reported to be in the range of 13–20 cases/million [12]. In contrast, data in children are scarce because most of the paediatric reports are based on single-centre and national retrospective cohort studies and also because vasculitis in children is extremely rare. The annual incidence of AAV reported in a recent retrospective multicentre French study including 66 children, the largest cohort in Europe, is ~0.5 per million children [13]. Higher incidence rates of up to 6.39 cases/million children/year were reported in a Canadian study [14]. Unlike in adults, where AAVs occur more frequently in male than in female patients, childhood AAVs are more common in female subjects and their onset peaks in the early second decade, with a median age at diagnosis of approximately 11–14 years [13]. These epidemiological characteristics were confirmed in the largest series of paediatric AAV reported so far, based on a North American multicentre study including 231 patients with MPA and GPA described by Cabral and colleagues. In this study, children with MPA were younger at diagnosis than those with GPA (median, 12 vs 14 years) [3]. Data on the epidemiology of EGPA are limited; in a literature review of 33 cases reported by Zwerina et al., childhood-onset EGPA was also more frequent in females, with an M:F ratio of 0.7, and the mean age at diagnosis was 12 years [15]. In terms of frequency, GPA is the most frequent, followed by MPA and EGPA. An examination of large databases (ARChiVe study) reveals that childhood-onset EGPA accounts for less than 2% of all cases of paediatric vasculitis [3].

### Pathogenesis

The pathogenesis of AAV is not entirely clear. Genetic susceptibility factors, environmental agents, as well as abnormalities in innate and adaptive immune responses appear to contribute to the development of AAV. A recent genome-wide association study (GWAS) of 2687 Northern European Caucasian patients with GPA and MPA suggested that GPA is associated with genetic variants within *HLA-DP*, *SERPINA1* (encoding α1-antitrypsin), and *PRTN3* (encoding PR3), whereas MPA is mainly associated with *HLA-DQ* [16]. Interestingly, these associations were stronger with ANCA specificity (PR3-ANCA and MPO-ANCA) than with the clinical diagnosis of GPA or MPA, suggesting that the differences in the genetic background of the two AAV forms were more closely related to the different ANCA profile than to the clinical diagnosis. This observation, together with the strong association of GPA with *PRTN3* (which encodes the autoantigen of PR3-ANCA) and with *SERPINA1* (encoding α1-antitrypsin, the major system able to catabolise PR3), suggested a central role of the autoantigen in the generation of ANCA and in the pathogenesis of AAV. [17] Several environmental agents are known to predispose to or trigger AAV; among these are air pollutants (especially silica), infections (*Staphylococcus aureus* and viral infections) [18, 19] and drugs (e.g. penicillamine, propylthiouracil, dapsone, cocaine adulterated with lemivasole) [20–23].

The pathogenesis is also based on defects in innate and adaptive immunity with a dysregulation of B cells, pathogenic production of ANCA, neutrophil activation and an imbalance between helper T cell and effector T cell responses. Neutrophils primed by infectious agents and also activated by ANCA are the main cells initiating endothelial cell and tissue damage, leading to inflammation of the vessel wall and- in GPA- granuloma formation [24]. Strong in vivo and in vitro evidence supports the pathogenic role of ANCA. ANCAs are predominantly immunoglobulin G (IgG) autoantibodies directed against constituents of neutrophil primary granules and lysosomes of monocytes. Animal models also exist that recapitulate the main phenotypic features of MPA using MPO-ANCA; on the other hand, the pathogenicity of PR3-ANCA has not clearly been demonstrated in animal models of vasculitis [24, 25]. Finally, a role of the complement alternative pathway has recently been demonstrated [24]. AAVs are traditionally considered pauci-immune diseases, i.e. diseases characterised by little or no immune deposits in the affected tissues. However, recent studies have shown that C3 deposition is not uncommon in patients with renal AAV, and that it is the expression of complement alternative pathway activation with consequent generation of chemotaxins (e.g. C3a, C5a) which are able to amplify the inflammatory response. Interestingly, serum C3 consumption is also a marker of adverse outcome in AAV. [26]

### Clinical manifestations

AAVs are systemic diseases. However, ‘limited’ forms may occur when they are confined to single organs. Table 3 reports the main demographic and clinical characteristics of two recently published series of paediatric AAV, one coming from North America [3] and one from France [13]. These series were selected because they represent different continents, were comprised of both GPA and MPA patients, provided detailed clinical phenotyping of the cases and their size was quite large. In the same table we also report the preliminary, unpublished results of a multicentre study conducted in Italy also including GPA and MPA patients. This study is still underway. Finally, the table reports the results of a retrospective literature review on paediatric EGPA [15].

**Table 3:** Main demographic and clinical characteristics of childhood-onset GPA, MPA and EGPA in two large published series (Cabral et al. and Sacri et al.), our unpublished Italian cohort and a literature review (Zwerina et al.)

	GPA			MPA			EGPA
	Cabral et al. [3]	Sacri et al. [13]	Italian cohort (unpublished)	Cabral et al. [3]	Sacri et al. [13]	Italian cohort (unpublished)	Zwerina et al. [15]
Total no. of patients	183	38	31	48	28	21	33
Female, no. (%)	113 (62)	21 (75)	22 (71)	35 (73)	34 (89)	11(52)	19 (58)
Caucasian, no. (%)	107 (59)	21 (75)	31 (100)	20 (42)	26 (68)	21 (100)	24 (73)
Median age (range) at diagnosis- *years*	14 (2–18)	12.8 (10.1-14.6)	14 (6–18)	12 (1–18)	11.2 (8.9-12.3)	11 (6–17)	13 (2–18)
Median time (range) to diagnosis- *months*	2.1 (0–73)	1.8 (0.8-5)	5 (0–60)	1.6 (0–39)	1.0 (0.6-8.1)	2.5 (1–24)	NA
Constitutional symptoms, no. (%)	160 (88)	23 (82)	21 (68)	41 (85)	29(78)	14 (67)	NA
Fever	97 (53)	19 (68)	16 (52)	25 (52)	18 (50)	11 (52)	NA
Fatigue	160 (88)	23 (82)	21 (68)	37 (77)	29 (78)	14 (67)	NA
Renal involvement, no. (%)	151 (83)	22 (78)	20 (65)	36 (75)	36 (95)	20 (95)	5/32 (16)
Pulmonary involvement, no. (%)	136 (74)	19 (68)	16 (52)	21 (44)	11 (29)	8 (38)	29 (88)
ENT, no. (%)	128 (70)	21 (75)	24 (78)	0 (0)	0 (0)	0 (0)	20 (77)
Eye involvement, no. (%)	78 (43)	6 (21)	14 (45)	15 (31)	3 (8)	0 (0)	NA
Cutaneous involvement, no. (%)	86 (47)	15 (54)	8 (26)	25 (52)	13 (34)	7 (33)	21 (66)
Gastrointestinal involvement, no. (%)	66 (36)	5 (18)	2 (6)	28 (58)	4 (11)	4 (19)	12 (40)
Musculoskeletal involvement, no. (%)	24 (14)	1 (4)	14 (45)	9 (19)	1 (3)	6 (29)	7 (22)
CNS involvement, no. (%)	36 (20)	1 (4)	2 (6)	10 (21)	2(5)	6 (29)	NA
Cardiovascular involvement, no. (%)	10 (5)	NA	2 (6)	3 (6)	NA	1 (5)	17 (55)

Common clinical presentation of paediatric AAV includes constitutional symptoms in more than 50% of patients. Fever, fatigue, anorexia and weight loss may precede the systemic organ manifestations and, as they are mostly non-specific findings, the diagnosis of AAV may be delayed. A delay in diagnosis is particularly found in patients with mild renal or otolaryngological manifestations [27].

In MPA, renal disease is the most frequent manifestation on presentation, followed by systemic features, musculoskeletal, cutaneous, lower respiratory tract and gastrointestinal involvement in the absence of granulomatous lesions [28]. As in adults, renal involvement in MPA is usually under the form of rapidly progressive glomerulonephritis. Lung involvement is less frequent but can be life-threatening in some cases, especially when alveolar haemorrhage occurs [29].

GPA is generally characterised by ear-nose-throat (ENT) involvement followed by constitutional symptoms, renal, lower respiratory tract, musculoskeletal and cutaneous involvement [28]. Frequent clinical features are: rhinitis, sinusitis, nasal crusts, epistaxis, otitis media, sensorineural or conductive hearing loss. In some cases, necrotising and granulomatous lesions cause destruction of the turbinates and nasal septum perforation. The most frequent lung lesions are nodules tending to excavation, single or multiple, sometimes confluent. In GPA, the frequency of renal disease ranges from 50 to 100% of children (in adults it is slightly less frequent) and the clinical pictures range from isolated urinary abnormalities (proteinuria, microscopic haematuria and/or blood red casts) to rapidly progressive glomerulonephritis, with consequent acute kidney injury.

In both GPA and MPA skin lesions can be present, such as palpable purpura, livedo reticularis, nodules, and non-specific rash. Up to one third of patients present with nonspecific musculoskeletal symptoms such as arthralgia, myalgia and sometimes arthritis. Neurological involvement is uncommon (seizures being the most frequent manifestation), with central nervous system involvement apparently being more frequent than peripheral neuropathy [3, 13].

These clinical features are usually shared by adult-onset and paediatric AAV and, as reported in Table 3, their frequency is quite homogeneous across the different cohorts. Thus, apparently, the clinical phenotypes of adult and paediatric forms are similar. There are, however, some differences: one of the most significant clinical discrepancies appears to be the frequency of subglottic stenosis. In childhood-onset AAV, the risk of subglottic stenosis is 5-fold higher than in adult patients; likewise, the risk of saddle nose deformity is also higher in children. Why this occurs is still unclear [30]. It is also interesting to note that peripheral neuropathy, which occurs in approximately one third of adult MPA or GPA patients [31], is extremely rare in paediatric AAV (2% in our Italian cohort), although it must be acknowledged that this clinical feature is not well described in other childhood AAV series. Also, the incidence of cardiovascular manifestations appears to be lower in paediatric AAV; it can be hypothesized that cardiovascular manifestations in AAV are the results of vasculitis but also of underlying atherosclerosis, which is obviously prevalent in the adult age.

Studies on the clinical phenotype of EGPA are, as already stated above, limited to few case reports or small case series and literature reviews. As in adult-onset EGPA, the paediatric form usually presents with asthma, eosinophilia, sinusitis, nasal polyps, and lung infiltrates. As compared with adult-onset EGPA, paediatric EGPA more frequently shows cardiomyopathy, which can account for the higher mortality rates described in paediatric cases. Importantly, publication bias of the reports (reviewed by Zwerina et al.) might have led to the inclusion of more aggressive cases. Other clinical features are skin lesions (usually purpura or urticarial skin rashes), which are found in up to two thirds of the cases, and gastrointestinal involvement. Interestingly, as in MPA and GPA, also in EGPA the frequency of neuropathy seems to be lower in paediatric than in adult-onset AAV. [15] (Table 3)

### Laboratory findings

ANCA positivity is common but not universal in childhood-onset AAV, as is in adult patients. In the largest cohort published to date, Cabral et al. [3] reported that PR3-ANCA and/or C-ANCA are positive in 67% of GPA and in 17% of MPA patients, whereas MPO-ANCA and/or P-ANCA are found in 55% of children with MPA and in 26% of those with GPA. ANCAs are more frequently negative in patients with limited disease forms (especially the ones confined to the upper airway tract). True dual ANCA-positivity is rare and raises suspicion of drug-induced vasculitis. As in adults, paediatric patients with EGPA are often ANCA negative; ANCA positivity, in fact, is found in 25% of paediatric EGPA (vs 35-40% of adults), usually with P-ANCA pattern or MPO-specificity [15].

Non-specific laboratory findings in childhood AAV include high acute-phase reactant levels (erythrocyte sedimentation rate and C-reactive protein); haematologic abnormalities, particularly anaemia, are also common.

### Histopathology: focus on renal lesions

The key histological feature of AAV is necrotising vasculitis, with few or no immune deposits, predominantly affecting small vessels. This can be associated with granulomatous lesions in GPA and eosinophil-rich (and often granulomatous) inflammation in EGPA. Tissue biopsies are crucial to make the diagnosis of AAV; based on the evidence of histologically compatible lesions, one can apply the classification criteria to define the different vasculitic syndromes. Notwithstanding, not all patients with AAV have diagnostic biopsies; this usually happens because some affected sites (e.g. upper airway tract) often yield non-diagnostic specimens, or because surrogate markers of vasculitis (e.g. subglottic stenosis, cavitated lung nodules) together with a clinically compatible phenotype and ANCA-positivity can lead to the diagnosis of AAV even in the absence of a biopsy [11].

However, when renal involvement is present in patients with suspected or confirmed AAV, renal biopsy still remains the gold standard for the diagnosis and is an important predictor of renal outcome. ANCA-associated glomerulonephritis is characterised by pauci-immune immunofluorescence, necrotising and crescentic lesions in the affected glomeruli at light microscopy and subendothelial oedema, microthrombosis and degranulation of neutrophils at electron microscopy. Although ANCA-associated glomerulonephritis is commonly described as a pauci-immune disease, some cases display focal deposition of immunoglobulins and complement fractions, especially C3 [26]; these patients have a poorer prognosis [32]. Glomerulonephritis in MPA differs from GPA-associated forms for the lack of granulomatous lesions and the more frequent occurrence of chronic lesions (e.g. glomerulosclerosis, fibrous crescents, interstitial fibrosis). From a clinical standpoint, MPA patients also tend to have more frequent and more severe renal involvement than those with GPA. In addition to glomerular involvement, renal disease in AAV also shows tubulointerstitial nephritis; tubular lesions are important predictors of outcome, especially in patients treated with B-cell depleting therapy [33].

In 2010, an international working group of renal pathologists proposed a histopathologic classification of ANCA-associated glomerulonephritis, predictive of long-term renal outcome in the adult population. The renal pathological features were divided into four distinct categories: focal, crescentic, sclerotic and mixed (Table 4). These categories predicted renal outcome in the short and long-term: patients with focal lesions had an excellent renal outcome, whereas renal survival was poor in the sclerotic class [34]. This classification system was confirmed not only in other adult cohorts [35], but also in paediatric studies. In 2014, Noone et al. used this histopathologic classification system in a retrospective study including 40 children with biopsy-proven ANCA-associated glomerulonephritis (MPA and GPA) followed for a median follow-up of 2.4 years. As in adults, the histopathological classification provided important prognostic information, with rapid progression to end-stage renal disease of patients in the sclerotic class, mild disease in the focal, and slow decline over 2 years in mixed and crescentic classes. Interestingly, in patients with renal relapse or progressive renal impairment, 80% of those with a crescentic pattern progressed to a sclerotic class on a second biopsy. These data suggest, for children with extensive sclerotic features, that aggressive immunosuppression is unlikely to result in renal function recovery, and that probably this should be avoided to limit short- and long-term adverse events. Conversely, patients with more florid lesions may require intensive treatment [36]. However, future prospective studies are needed for better optimisation of treatment based on pathologic features.

**Table 4:** Berden’s classification [34] of ANCA associated glomerulonephritis

Class	
Focal	≥50% normal glomeruli
Crescentic	≥50% glomeruli with cellular crescents
Mixed	<50% normal, < 50% crescentic, < 50% globally sclerotic glomeruli
Sclerotic	≥50% globally sclerotic glomeruli

### Disease activity scoring

The Birmingham Vasculits Activity Score (BVAS) is a comprehensive multisystem tool for standard assessment of systemic vasculitis, used in adult clinical trials [37]. The latest version of this score is the BVAS V3 [38]. The original BVAS was subsequently modified and is recommended for use in AAV clinical trials as part of the Outcome Measures in Rheumatology (OMERACT) core set of outcome measures. In adults, the Vasculitis Damage Index (VDI) is also used for the standardised clinical assessment of damage in systemic vasculitides [39]. A paediatric tool based on modifications to the BVASv3, the Paediatric Vasculitis Activity Score (PVAS), was developed in the 2012 by Dolazalova et al., but needs further validation in independent data set. The score ranges from 0 to 63 with higher scores indicating higher disease activity [40, 41]. The Paediatric Vasculitis Damage Index (PVDI) is based on a modified version of the adult VDI tool; the score ranges from 0 to 73, with higher scores indicating increased organ damage. Despite the fact that the paediatric version is not yet formally validated, it was used in a retrospective experience on childhood-onset EGPA in the UK [42].

### Treatment and outcome

Advances in the treatment of systemic vasculitis have significantly improved patient outcomes in the last decades. The introduction of glucocorticoids and cyclophosphamide (CYC) dramatically improved the prognosis of affected patients, with approximately 80% surviving at 5 years compared with a 1-year mortality of 80% in untreated adult patients [43]. In the absence of any controlled trials in paediatric AAV, treatment remains based on adult data and is structured on a sequential approach comprising remission-induction and remission-maintenance phases.

For remission-induction of organ- or life-threatening AAV in adults, the EULAR-ERA EDTA recommendations indicate treatment with a combination of glucocorticoids and either CYC or rituximab [44]. High-dose glucocorticoids and CYC (either oral or intravenous) for 3–6 months are considered the gold standard of treatment also in children. As an alternative to oral daily CYC, the intermittent intravenous pulse regimen (15 mg/kg/pulse, maximum 1.2 g, every 2–3 weeks for 3–6 months) reduces cumulative CYC exposure [45–47], and is therefore routinely used in children. With regards to rituximab, two randomised trials in adult patients demonstrated its non-inferiority to CYC for remission induction in AAV [48, 49]. The EULAR-ERA EDTA recommendations suggest RTX be considered for treatment of children not responding to conventional treatments and for patients with relapsing disease [50]. As opposed to CYC, rituximab does not impair fertility and carries an apparently lower malignancy risk. This is why, despite the lack of solid data in children, its use in the paediatric population has substantially increased; a recent study of children hospitalised for AAV in the United States has shown a shift from the use of CYC to the use of rituximab [51]. However, larger studies with long-term follow-up are needed to establish the efficacy and safety of rituximab in children. Basu et al. showed the efficacy of a sequential regimen of rituximab induction followed by mycophenolate mofetil maintenance (2–3 years) in 11 children with MPA. After a median follow-up of 20.9 months, 73% of patients were off steroids (but still on mycophenolate) and 82% never relapsed. Mycophenolate mofetil has been tested also as induction therapy for AAV in the MYCYC trial (clinicaltrials.gov NCT00414128), a recently completed study whose results are awaited soon; MYCYC also included paediatric patients. Other small, non-randomised studies used mycophenolate for induction in adults, with generally positive results [52]. In AAV patients with limited forms or non-organ threatening manifestations, remission can be obtained with methotrexate, which proved of equal efficacy to CYC in adult patients with this disease subset enrolled in the NORAM trial [53]. On the other hand, very severe cases, presenting with rapidly progressive renal failure or alveolar haemorrhage, may benefit from the addition of plasma exchange to standard CYC-based therapies [54]. In the largest published cohort of paediatric MPA and GPA, Cabral et al. report the use of plasma exchange in 21% of the cases [3].

Once remission has been induced, maintenance with drugs other than CYC is recommended. Azathioprine (AZA) is the mainstay of remission maintenance since the publication of the CYCAZAREM trial, which showed that its ability to prevent relapses was similar to that of CYC [55]. Other options are available such as methotrexate [56]. Mycophenolate mofetil proved inferior to AZA in a randomised trial, therefore it is not recommended as first-line maintenance therapy [57]. Finally, a recent trial compared low-dose rituximab to azathioprine for remission maintenance: rituximab-treated patients had significantly fewer relapses than the AZA-treated ones [58]. Remission maintenance should be continued for at least 18–24 months.

AAVs are usually considered chronic-relapsing disorders; at least in adults, the frequency of relapses is significantly higher in GPA or PR3-ANCA positive patients than in those with MPA or with MPO-ANCA. As long-term follow-up studies of large cohorts are lacking, the precise frequency of relapse in paediatric AAV is still unclear. In a retrospective French study of 35 AAV patients followed for a median of 8 years, relapse rates ranged from 33% in MPA to 50% in EGPA and 83% in GPA [59]. This underlines the importance of a careful follow-up and a prolonged remission maintenance therapy.

AAV patients accumulate damage over time, as a result of both the disease itself and treatment-related toxicity [60]. The risk of infection and infertility is significant; moreover, particularly in children exposed to high cumulative CYC doses, surveillance for neoplasms is mandatory. Common *sequelae* of AAV include saddle nose deformity and subglottic stenosis (in GPA), asthma and chronic sinusitis (particularly in EGPA), peripheral neuropathy with sensori-motor deficits, and particularly chronic renal failure. Renal disease is an important determinant of long-term prognosis and morbidity in paediatric AAV; it is more frequent and severe in MPA than in GPA, whereas it is usually mild and tends not to progress to end-stage renal disease in EGPA. In a study reviewing seven reports of GPA and MPA in children, the rate of end-stage renal disease ranged between 29% and 40% in MPA, whereas it was around 10% in GPA [60]. In a single-centre, cohort study of 40 well-characterised patients with ANCA-associated glomerulonephritis, after a median follow-up of 2.4 years, 33.3% progressed to end-stage renal disease whereas 42.4% developed chronic kidney disease not necessitating renal replacement therapy [36]. As reported above, most of the studies that considered renal histology at diagnosis showed that the histological category predicted renal survival, with the sclerotic category portending a poorer outcome as compared to the other classes. In patients who develop end-stage renal disease, renal transplantation is a feasible treatment option, although data on children are still limited.

Fortunately, mortality rates are low in paediatric AAV and usually do not exceed 5-10% [36, 59]. Childhood-onset EGPA patients only seem to have a poorer survival (mortality rates of 15-18%), which is probably due to the relatively high frequency of cardiac involvement [17, 42]; these data, however, must be taken with caution as they are based on case reports or small case series.

## Conclusions

AAVs are rare diseases in childhood, they may be severe and organ- or life-threatening. Their clinical presentation is often similar to that of adult patients, although some differences exist. Renal involvement drives the prognosis of paediatric AAV, with end-stage renal disease requiring dialysis or transplantation being one of the most severe long-term complications. The treatment of paediatric AAV still relies on the studies performed in adults; induction with CYC has been the mainstay of initial therapy, although the efficacy of RTX demonstrated in adults has made this treatment an attractive option in children as well. Although the long-term outcome of paediatric AAV seems more favourable than that observed in adults, larger multicentre studies are needed to better define the prognosis of these patients.
